# The cognitive connectome in dementia with lewy bodies undergoes early alterations already at the mild cognitive impairment stage

**DOI:** 10.1038/s41598-025-24157-7

**Published:** 2025-10-23

**Authors:** Roraima Yanez-Perez, Annegret Habich, Jon B. Toledo, José Barroso, Daniel Ferreira

**Affiliations:** 1 Division of Clinical Geriatrics, Centre for Alzheimer Research, Department of Neurobiology, Care Sciences, and Society, Karolinska Institutet, Stockholm, Sweden; 2 Department of Clinical Psychology, Psychobiology and Methodology, Faculty of Psychology, University of La Laguna, Canary Islands, Spain; 3 University Hospital of Psychiatry and Psychotherapy, University of Bern, Bern, Switzerland; 4 Stanley H. Appel Department of Neurology, Nantz National Alzheimer Center, Houston Methodist Hospital, Houston, TX USA; 5 Facultad de Ciencias de la Salud, Universidad Fernando Pessoa Canarias, Las Palmas, Spain; 6 Division of Clinical Geriatrics, Centre for Alzheimer Research, Department of Neurobiology, Care Sciences and Society, Karolinska Institutet, NEO floor 7th, Huddinge, 141 83 SE Sweden

**Keywords:** Cognition, Dementia with Lewy bodies, Mild cognitive impairment, Graph theory, Network analysis, Connectome, Cognitive ageing, Dementia, Neurodegenerative diseases

## Abstract

Cognitive impairment is required to diagnose mild cognitive impairment with Lewy bodies (MCI-LB). However, associations of impairments across cognitive domains remain unclear. In this cross-sectional study, we investigated these associations by assessing the cognitive connectome of MCI-LB patients compared with healthy controls (HC), mild cognitive impairment due to Alzheimer’s disease (MCI-AD), and dementia with Lewy bodies (DLB). Using the National Alzheimer’s Coordinating Center database, we built cognitive connectomes for MCI-LB (*n* = 88), HC (*n* = 3703), MCI-AD (*n* = 1789), and DLB (*n* = 104) by correlating 24 cognitive measures. We compared global and nodal network measures of centrality (importance of cognitive measure), integration (communication across cognitive measures), and segregation (specialisation of cognitive measures) between groups. For global measures, MCI-LB showed lower segregation than HC, with no significant differences from MCI-AD, and lower integration) and higher segregation than DLB. For nodal measures, MCI-LB compared with HC and MCI-AD showed differences in executive and memory measures, respectively. MCI-LB showed several nodal differences compared with DLB, involving executive, processing speed/attention, and language measures. Our findings suggest that MCI-LB involves early changes in the cognitive connectome, particularly reduced segregation that becomes more pronounced at the DLB stage and shows overlap with MCI-AD, offering insights into cognitive impairment in MCI-LB.

## Introduction

Dementia with Lewy bodies (DLB) accounts for up to 24% of all neurodegenerative dementia cases^1^. DLB is characterized by progressive cognitive decline together with its core clinical features of parkinsonism, rapid eye movement (REM) sleep behavior disorder (RBD), cognitive fluctuations, and visual hallucinations^2^. Prodromal symptoms can occur 15 years or more before people with prodromal DLB progress to overt DLB^3,4^. Mild cognitive impairment with Lewy bodies (MCI-LB) is the most common form of prodromal DLB^5^.

So far, research on cognition in MCI-LB has focused on comparing the cognitive performance of MCI-LB patients with healthy controls (HC) or patients with other forms of MCI, such as MCI due to Alzheimer’s disease (MCI-AD). According to a recent meta-analysis, MCI-LB patients perform worse than HC in several cognitive domains, with the largest effect size for processing speed/attention^6^. MCI-LB patients presented lower performance in attention/executive functions and processing speed/attention and higher performance in memory than MCI-AD patients^6^. Compared with DLB, MCI-LB patients perform better in several cognitive domains, with the largest effect size for attention/processing speed and attention/executive functions^6^. Although univariate approaches can provide information on impairments in distinct cognitive tests, they do not consider how impairments across diverse cognitive tests are interconnected. More sophisticated multivariate analyses are needed to characterize the complex associations across cognitive tests in MCI-LB^7^. However, multivariate studies investigating associations among cognitive measures are scarce in MCI-LB. To our knowledge, only one study analysed associations among specific cognitive measures in MCI-LB^8^. The authors showed that processing speed mediated the performance in visuospatial working memory and memory in MCI-LB patients^8^. However, the complex associations among cognitive domains in MCI-LB remain largely unknown.

The “cognitive connectome”^9^ is a novel concept that represents the intricate organization and associations among cognitive domains in a comprehensive manner. In contrast to conventional multivariate approaches, which summarize associations, the connectome/network approach preserves their topology, providing a more differentiated view of impairments across different cognitive domains^10^. Network analyses can identify how central specific cognitive measures relate to the connectome and give insights into the integration and segregation of such connectome^11^. While the cognitive connectome has been investigated in normal aging and various pathological conditions such as epilepsy, acquired brain injury, vascular encephalopathy, Parkinson’s disease, MCI-AD, and AD^9,11–17^, only one study has investigated the cognitive connectome in DLB^18^. In that study, we demonstrated that the cognitive connectome of patients with DLB shows a loss of segregation, leading to a loss of cognitive specialization compared with the connectomes of HC and patients with AD^18^. These findings raise the critical question of whether alterations in the cognitive connectome appear already in prodromal stages of DLB.

In the current study, we investigated the cognitive connectome of patients with MCI-LB by pursuing three specific aims. First, we aimed to characterize the cognitive connectome of patients with MCI-LB through a comparison with HC. Second, we compared the cognitive connectome of MCI-LB and MCI-AD to understand potential differences between these two common MCI groups. Third, we compared the cognitive connectome of MCI-LB and DLB to investigate changes in the cognitive connectome during the disease progression. We hypothesized modest differences between MCI-LB and HC groups, likely involving processing speed/attention and executive domains. Moreover, we hypothesized modest differences between MCI-LB and MCI-AD, likely involving memory, processing speed/attention, and executive domains. Finally, we hypothesized that the cognitive connectome of MCI-LB would show alterations of intermediate severity between HC and DLB patients.

## Results

### Cohort characteristics

Demographic and clinical characteristics of the four groups are reported in Table 1. MCI-LB did not differ in age from HC and DLB patients, while MCI-LB patients were statistically significantly younger than MCI-AD patients. There were significantly more men in MCI-LB than in both HC and MCI-AD. MCI-LB completed more years of education than HC and MCI-AD. MCI-LB and MCI-AD did not differ in the CDR total score.

Univariate analyses (ANCOVAs) show that MCI-LB performed significantly worse than HC across all cognitive domains. Moreover, compared with MCI-AD, MCI-LB performed worse in visuoconstructive and processing speed/attention domains, while MCI-LB performed better in visual and verbal episodic memory and orientation domains. Additionally, compared to DLB, MCI-LB performed better in all the studied cognitive domains.

**Table 1:** Key demographic and clinical characteristics.

	MCI-LB (*N* = 88)	HC (*N* = 3703)	MCI-AD (*N* = 1789)	DLB (*N* = 104)	MCI-LB vs. HC	MCI-LB vs. MCI-AD	MCI-LB vs. DLB
	Mean (SD)	Mean (SD)	Mean (SD)	Mean (SD)	*p*-value	*p*-value	*p-*value
Age, years min-max	70.6 (7.4) 51–87	72.2 (9.2) 45–101	74.5 (8.4) 48–99	71.9 (8.5) 45–88	0.1	< 0.001	0.3
Sex, men (%)	89%	43%	51%	86%	< 0.001	< 0.001	0.7
Education, years	16.7 (2.6)	16.0 (2.9)	16.0 (2.9)	16.3 (3.1)	< 0.05	< 0.05	0.4
CDR-total, median (interquartile range)	0.5 (0.5–0.5)	0 (0–0)	0.5 (0.5–0.5)	1 (0.5–1.5)	< 0.001	0.3	< 0.001
Cognitive fluctuations, present (%)	29%	1%	1%	60%	< 0.001	< 0.001	< 0.001
Visual hallucinations, present (%)	24%	1%	1%	43%	< 0.001	< 0.001	< 0.01
Probable RBD, present (%)	66%	1%	1%	65%	< 0.001	< 0.001	1
Parkinsonian signs, present (%)	74%	3%	1%	82%	< 0.001	< 0.001	0.3
Cognitive domains							
Visuoconstructive functions	−0.8 (1.1)	0 (0.8)	−0.4 (0.9)	−1.8 (1.8)	< 0.001	< 0.01	< 0.001
Visual and verbal episodic memory	−0.7 (0.7)	0 (0.6)	−1 (0.8)	−1.5 (0.9)	< 0.001	< 0.001	< 0.001
Executive functions	−0.3 (0.5)	0 (0.5)	−0.3 (0.5)	−0.9 (0.8)	< 0.001	0.2	< 0.001
Processing speed and attention	−0.7 (1.1)	0 (0.7)	−0.3 (0.9)	−2.1 (1.6)	< 0.001	< 0.01	< 0.001
Language	−0.3 (0.8)	0 (0.8)	−0.4 (1)	−1 (1.1)	< 0.001	0.7	< 0.001
Orientation	−0.4 (1.7)	0 (1)	−1.2 (2.5)	−3.1 (4.2)	< 0.05	< 0.001	< 0.001

Mean (standard deviation) reported in the table, if not otherwise specified. Cognitive domains expressed as averaged z-scores. For CDR, there was missing data for 4 MCI-LB, 411 HC, 129 MCI-AD, and 1 DLB. For cognitive fluctuations, there was missing data for 1 MCI-LB, 1 HC, 47 MCI-AD, and 2 DLB. For visual hallucinations, there was missing data for 1 HC, and 2 MCI-AD. Probable RBD data was missing for 1 MCI-LB, 7 HC, 15 MCI-AD, and 3 DLB. Comparisons were established a priori for MCI-LB vs. HC, MCI-LB vs. MCI-AD and MCI-LB vs. DLB. Abbreviations: CDR, Clinical Dementia Rating scale; DLB, Dementia with Lewy bodies; HC, healthy controls; MCI-AD, mild cognitive impairment due to Alzheimer’s Disease; MCI-LB, mild cognitive impairment with Lewy bodies; RBD, rapid eye movement sleep behavior disorder.

### Weighted correlation matrices

**Fig. 1:**
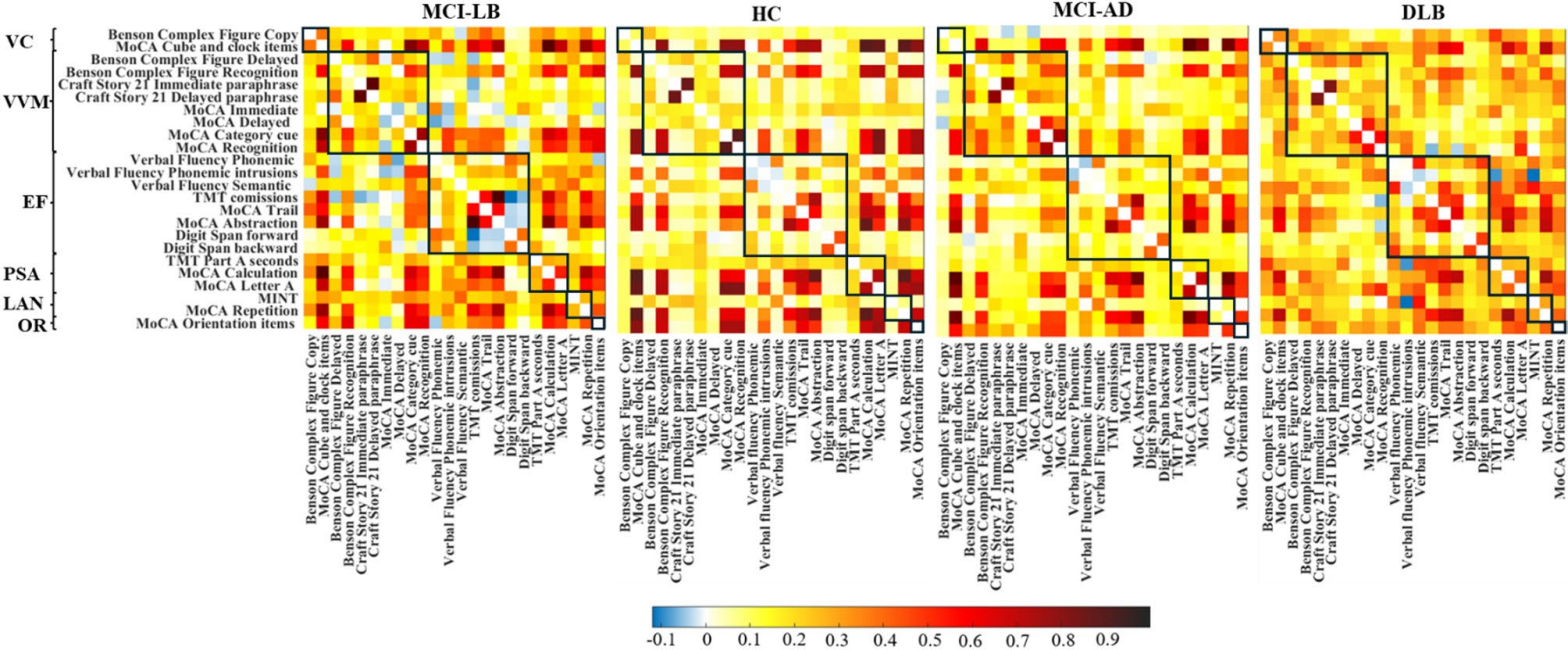
Correlation matrices of MCI-LB, HC, MCI-AD, and DLB groups. Negative correlations were observed in all groups. The color bar shows the same Spearman’s correlation coefficient range for all four groups. Abbreviations: DLB, dementia with Lewy bodies; EF, executive functions; HC, healthy controls; LAN, language; MCI-AD, mild cognitive impairment due to Alzheimer’s disease; MCI-LB, mild cognitive impairment with Lewy bodies; MINT, Multilingual Naming Test; MoCA, Montreal Cognitive Assessment; OR, orientation; PSA, processing speed/attention; TMT: Trail Making Test; VC, visuoconstructive functions; VVM, visual and verbal episodic memory.

Cognitive connectomes of the four groups are depicted in Fig. 1. Visual inspection of the cognitive connectome in MCI-LB showed a predominance of weak to moderate correlations within and between the six cognitive domains. 10% of the correlations were negative. In contrast, the cognitive connectome of HC was characterized by both weak and strong correlations within and between cognitive domains (small-world topology), with only 1% of negative correlations. The cognitive connectome of MCI-AD was similar to that of MCI-LB, mainly exhibiting weak to moderate correlations within and between cognitive domains, with 1% of negative correlations. However, we observed some differences, such as executive functions showing the most negative correlations within this domain’s tests and with the memory domain tests in MCI-LB, as compared with MCI-AD. Finally, visual inspection of the cognitive connectome of DLB showed the most homogeneous pattern of weak correlations within and between cognitive domains, with 2% of negative correlations.

Figure 1. Correlation matrices of MCI-LB, HC, MCI-AD, and DLB groups. Negative correlations were observed in all groups. The color bar shows the same Spearman’s correlation coefficient range for all four groups. Abbreviations: DLB, dementia with Lewy bodies; EF, executive functions; HC, healthy controls; LAN, language; MCI-AD, mild cognitive impairment due to Alzheimer’s disease; MCI-LB, mild cognitive impairment with Lewy bodies; MINT, Multilingual Naming Test; MoCA, Montreal Cognitive Assessment; OR, orientation; PSA, processing speed/attention; TMT: Trail Making Test; VC, visuoconstructive functions; VVM, visual and verbal episodic memory.

### Global network measures

**Fig. 2:**
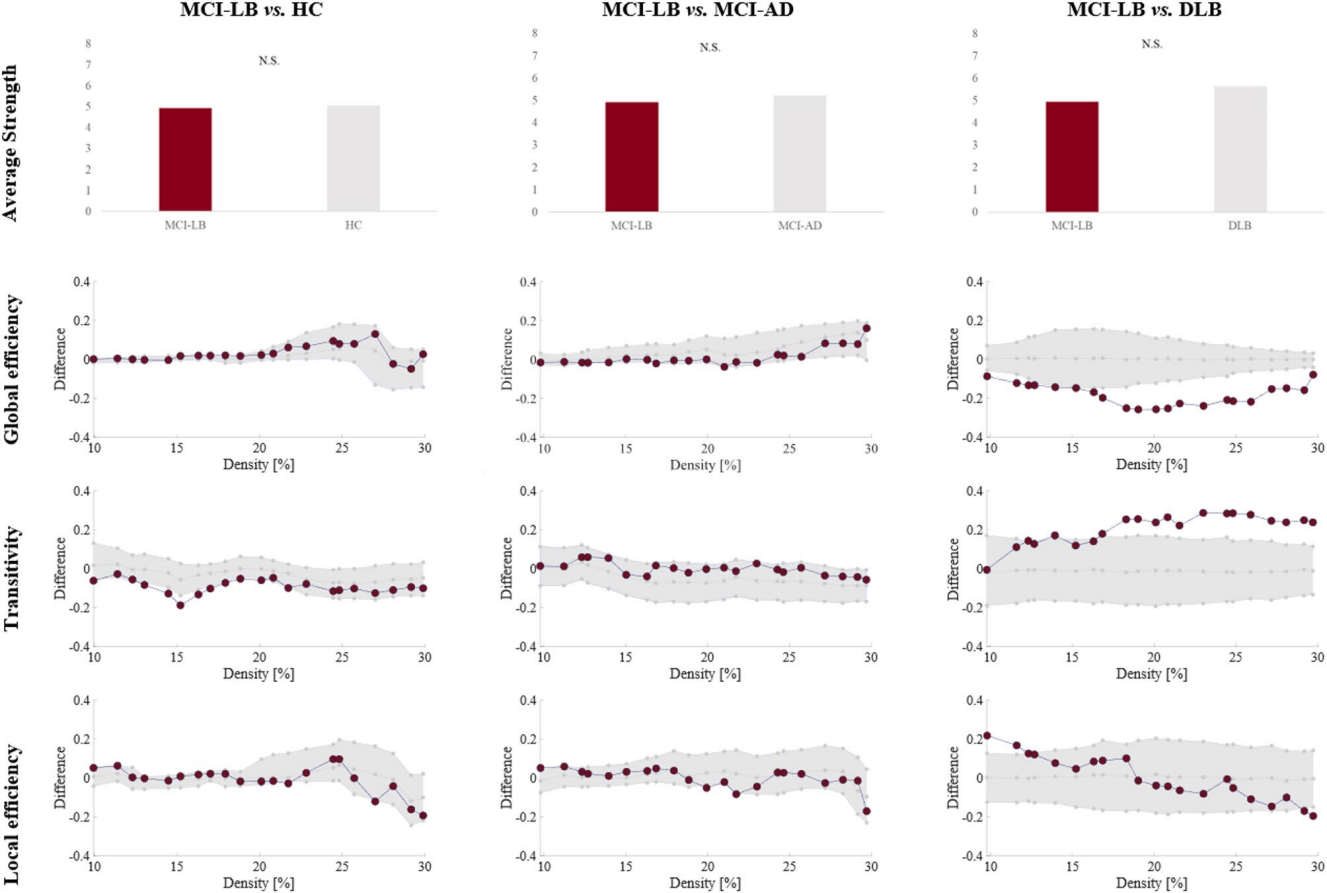
Differences between MCI-LB and the HC, MCI-AD and DLB groups in global network measures. For global efficiency, transitivity, and local efficiency, densities are displayed on the x-axis from min = 10% to max = 30%, in steps of 1%. Group differences are displayed on the y-axis. The grey area illustrates group differences with 95% confidence intervals. Red dots illustrate observed group differences. Negative differences indicate lower value in MCI-LB compared to HC, MCI-AD or DLB. Positive differences indicate higher values in MCI-LB compared to HC, MCI-AD or DLB. Between-group differences in global efficiency, transitivity, and local efficiency are significant when at least five consecutive red circles fall out of the grey-shaded area. Average strength tested on weighted matrices and therefore densities are not available. Abbreviations: DLB, dementia with Lewy bodies; HC, healthy controls; MCI-AD, mild cognitive impairment due to Alzheimer’s disease; MCI-LB, mild cognitive impairment with Lewy bodies; N.S., non-significant.

Quantitative differences in global network measures between MCI-LB patients and HC, MCI-AD, and DLB groups are illustrated in Fig. 2. Compared with the HC group, MCI-LB patients exhibited a statistically significant lower transitivity. No differences arose between MCI-LB and MCI-AD patients. Compared with the DLB group, MCI-LB patients showed lower global efficiency and higher transitivity. All these significant differences were replicated in leave-five-out analyses, as well as when using groups of equal size (data not shown).

Figure 2. Differences between MCI-LB and the HC, MCI-AD and DLB groups in global network measures. For global efficiency, transitivity, and local efficiency, densities are displayed on the x-axis from min = 10% to max = 30%, in steps of 1%. Group differences are displayed on the y-axis. The grey area illustrates group differences with 95% confidence intervals. Red dots illustrate observed group differences. Negative differences indicate a lower value in MCI-LB than in HC, MCI-AD, or DLB. Positive differences indicate higher value in MCI-LB than in HC, MCI-AD, or DLB. Between-group differences in global efficiency, transitivity, and local efficiency are significant when at least five consecutive red circles fall out of the grey-shaded area. Average strength was tested on weighted matrices and, therefore, densities are not available. Abbreviations: DLB, dementia with Lewy bodies; HC, healthy controls; MCI-AD, mild cognitive impairment due to Alzheimer’s disease; MCI-LB, mild cognitive impairment with Lewy bodies; N.S., non-significant.

### Nodal network measures

**Fig. 3:**
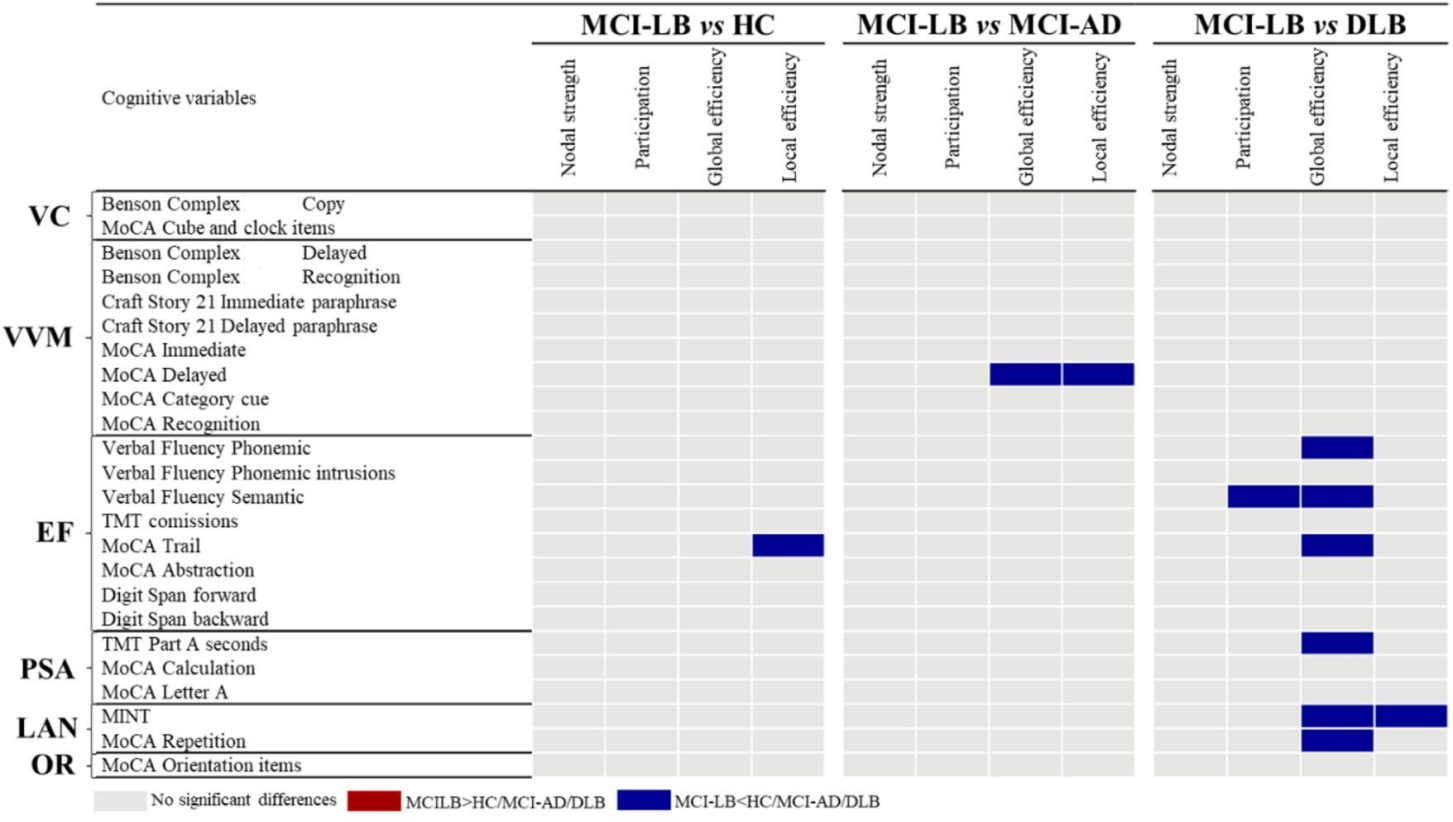
Summary of group differences in nodal network measures. False discovery rate (FDR) adjustment at *p* ≤ 0.05 (two-tailed) in all comparisons. Abbreviations: DLB, dementia with Lewy bodies; EF, executive functions; HC, healthy controls; LAN, language; MCI-AD, Mild Cognitive Impairment due to Alzheimer’s dsease; MCI-LB, mild cognitive impairment with Lewy bodies; MINT, Multilingual Naming Test; MoCA, Montreal Cognitive Assessment; OR, orientation; PSA, processing speed/attention; TMT: Trail Making Test; VC, visuoconstructive functions; VVM, visual and verbal episodic memory.

Differences in nodal measures between MCI-LB patients and HC, MCI-AD, and DLB groups are depicted in Fig. 3. We observed scarce differences in the comparisons between MCI-LB and HC and between MCI-LB and MCI-AD. On the contrary, most differences arose in the comparison between the MCI-LB and DLB groups.

Compared to HC, the MCI-LB group showed a lower local efficiency in an executive measure (MoCA Trail).

Compared to MCI-AD, the MCI-LB group showed a lower global and local efficiency in a verbal memory measure (MoCA Delayed).

The global efficiency measure captured most of the differences in the comparison between MCI-LB and DLB. Specifically, MCI-LB patients showed a lower global efficiency in executive, processing speed/attention, and language measures. Additionally, the MCI-LB group showed a lower local efficiency in a language measure (MINT), and a lower participation in an executive function measure (Verbal fluency Semantic).

Since the local efficiency captured significant differences for the three diagnostic comparisons, we replicated those results with leave-five-out analyses, showing the stability of the findings (data not shown).

## Discussion

We investigated the cognitive connectome of MCI-LB patients using network analyses on cognitive measures and compared it with the cognitive connectome of HC individuals and patients with MCI-AD and DLB. Our results showed modest alterations in the cognitive connectome of MCI-LB compared with HC in global and nodal segregation features. The cognitive connectomes of MCI-LB and MCI-AD were mostly similar, exhibiting only nodal differences in one memory-related cognitive variable. In contrast, the cognitive connectomes of MCI-LB and DLB showed significant differences at global and nodal levels of integration, segregation, and centrality features, with alterations becoming more pronounced in DLB.

### Characterization of cognitive connectome: MCI-LB vs. HC

Our first aim was to characterize the cognitive connectome of patients with MCI-LB through a comparison with a HC group. Visual inspection revealed that the cognitive connectome of MCI-LB patients was characterized by weak to moderate correlations within and between cognitive domains. At the same time, HC exhibited a small-world topology combining strong and weak correlations. Small-world topologies are characterized by the balance between segregation and integration features of the network, which is thought to promote an efficient transfer of information throughout the network^19^. In our study, our findings suggest that the loss of small-worldness in MCI-LB was primarily due to a lower transitivity than in HC in the global network analysis. A lower transitivity indicates that neighbouring nodes correlate more sparsely, potentially reflecting a loss of segregation^20,21^ in the cognitive connectome of MCI-LB. This loss of segregation suggests a loss of cognitive specialization in the connectome and a more random topology, which may lead to a less efficient transfer of information^22^ across the connectome of MCI-LB patients. Such a shift from an optimal small-world toward a more random topology likely reduces the efficiency of information exchange between cognitive domains, possibly resulting in the slowing in processing speed observed in MCI-LB compared with HC in our study and in a previous meta-analysis^6^. In connection with this interpretation, Ciafone and colleagues^8^ performed hierarchical multiple regressions, finding that processing speed accounted for the variance in visuospatial working memory and verbal memory performance in MCI-LB. Moreover, our results are in line with our previous study on DLB individuals where we found a loss of transitivity at the dementia stage, in addition to a loss of local efficiency and a higher global efficiency in DLB compared with HC^18^.

For nodal network measures, we observed only one statistically significant difference between MCI-LB and HC, involving a lower local efficiency in the MoCA-Trail. The MoCA Trail is a measure of both executive and attentional functioning. Although previous studies suggest that a lower efficiency is related to a higher cognitive performance^9,23^, in this case, the lower local efficiency in this executive/attentional measure might reflect the frequent lower performance of MCI-LB patients on Trail-like tests observed in univariate studies^24,25^. This finding is consistent with our hypothesis, as we expected to find differences in executive functions and attention when comparing MCI-LB and HC. The scarcity of significant differences between MCI-LB and HC in nodal measures is in line with a previous study on the cognitive connectome of an unspecific MCI group^11^ and two neuroimaging studies on functional and structural networks in MCI-LB^26,27^.

### Differentiation of cognitive connectomes: MCI-LB vs. MCI-AD

Our second aim was to compare the cognitive connectomes of MCI-LB and MCI-AD to understand potential differences between these two common MCI groups. Visual inspection revealed a somewhat similar pattern of weak to moderate correlations in both MCI groups. Nevertheless, we observed subtle visual differences such as the predominance of negative correlations in executive functions in MCI-LB, as compared with MCI-AD, which could reflect a more heterogeneous performance in MCI-LB. The quantitative analyses showed only two statistically significant differences in nodal network measures, while no significant differences were observed in global measures. Specifically, the MCI-LB group exhibited lower global and local efficiency in a verbal memory measure with an important retrieval component (i.e., MoCA Delayed). As a lower efficiency in the cognitive connectome has previously been noted in individuals with high cognitive performance^9,23^, our results may reflect the better performance in memory in MCI-LB compared with MCI-AD observed in our study in the univariate analysis and in a previous meta-analysis^6^. The lack of significant differences in global network measures may be explained by the fact that the cognitive connectome of MCI-AD or amnestic MCI patients also presents alterations in integration and segregation features compared with HC^14,16^. Ferguson and colleagues^14^ found more edges among the different cognitive domains in amnestic MCI patients compared with HC, as well as fewer modules in the modular analysis. These findings may be suggestive of a loss of segregation in amnestic MCI. In our current study, we also found the loss of network segregation to be characteristic of a different MCI population, i.e., MCI-LB. Therefore, further research is warranted to conclude the specificity of global alterations in the cognitive connectome of various types of MCI.

### Progression in cognitive connectomes: MCI-LB vs. DLB

Our third aim was to compare the cognitive connectome of MCI-LB and DLB, as the cognitive connectome might change along the progression of the disease. We observed more significant differences when comparing MCI-LB with DLB than when comparing MCI-LB with HC or MCI-AD. This might be related to the fast progression to dementia observed in MCI-LB patients in longitudinal studies^28^. For global network measures, the MCI-LB group showed a lower global efficiency and a higher transitivity than the DLB group. This pattern reflects a lower integration and higher segregation at the global network level in MCI-LB. Taken together with the higher performance of MCI-LB in all cognitive domains (univariate analysis) compared with DLB, this pattern suggests a progressive dedifferentiation of the cognitive functioning^29^. In other words, the cognitive connectome of MCI-LB seems to be at an intermediate severity level between that of HC and DLB, showing a loss of cognitive specialization that becomes more pronounced in DLB^18^.

For nodal network measures, the most prominent findings when comparing MCI-LB and DLB emerged in the global efficiency measure. We observed lower nodal global efficiency in executive, processing speed/attention, and language measures in MCI-LB. The lower nodal global efficiency in the MCI-LB group may reflect a more heterogeneous performance in the executive, processing speed/attention, and language measures compared with DLB, where most of the cognitive measures within those cognitive domains might have declined. At the same time, the emergence of significant differences, especially in executive and processing speed/attention domains, reflects the cognitive profile of MCI-LB^6,30^, with deficits in these domains becoming more pronounced in the clinical transition from MCI-LB to the stage of overt DLB. Other less prominent nodal findings emerged in local efficiency and participation measures, with MCI-LB showing a lower local efficiency in a language measure involving naming by visual confrontation (MINT) and lower participation in an executive measure involving semantic fluency (Verbal fluency Semantic). The lower local efficiency in language in MCI-LB compared with DLB may reflect a better performance in the naming task. Although some studies have reported an impaired performance in picture naming in MCI-LB^24^, this impairment might be more marked at the dementia stage. A meta-analysis found large effect sizes with better performance in MCI-LB vs. DLB in a language composite score that included naming tasks^6^. The participation result in semantic fluency indicates that this measure is more central in the cognitive connectome of DLB patients. This higher centrality might reflect a lower performance in DLB patients vs. MCI-LB, as cognitive measures that are more severely impaired tend to be more central in the cognitive connectome. The cited meta-analysis found large effect sizes for the lower performance of DLB in a composite score including a semantic fluency task, compared with MCI-LB^6^. Generally, our findings suggest that cognitive measures that are increasingly impaired during the disease progression tend to be more central in differentiating the cognitive connectomes across the different clinical stages. The lack of statistically significant results in other relevant cognitive domains such as visuoconstructive functions, might be related to the fact that our DLB patients are at a mild stage of dementia (i.e. median CDR: 1), thus the associations between alterations in some cognitive measures might be similar to those at the MCI-LB stage.

## Limitations

This study has some limitations. Data availability was not uniform across cognitive measures and we had to exclude four cognitive measures with missing values, including variables from the Trail Making Test-Part B and the MINT after cues. Since these tests tap into executive functions and language, we cannot exclude that retaining them in the connectome would have increased the differences between the MCI-LB and HC groups, as missing data may partly reflect difficulties to complete the task. Some cognitive domains were represented by less variables than other domains, which may have led to their underrepresentation. Most of our interpretations and conclusions are formulated in terms of cognitive domains to alleviate this limitation. The potential use of the same cognitive measures to diagnose and construct cognitive connectomes may have introduced certain circularity in our results. This could lead to a predominance of differences in cognitive tests used to diagnose MCI-LB. However, diagnosis was based on clinical judgement of many measures other than cognitive tests, which mostly mitigates this limitation. Indeed, we did not obtain significant differences in global network measures for the comparison between MCI-LB and MCI-AD, and only one nodal network measure was significant, which likely reflects the lack of circularity issues. We did not control for possible AD co-pathology in our MCI-LB group, which might have contributed to the lack of significant results between MCI-LB and MCI-AD. In manifest DLB, patients with DLB who have AD co-pathology have poorer performance in memory and orientation domains than patients with DLB without AD co-pathology^31^. However, it is still unclear to what extent AD co-pathology may contribute to specific cognitive domains in the earlier stage of MCI-LB. Similarly, while NACC has implemented harmonized data collection across participating centres, minimising rater variability across centres, potential confounders such as comorbidities and medications could influence cognitive performance of participants and the resulting cognitive connectomes. To reduce confounding, we applied exclusion criteria for major neurological comorbidities. Due to information on medication intake not being available for all participants, this variable was not included as a covariate. Furthermore, the number of pathologically confirmed MCI-LB cases in our cohort was low, a common limitation in clinical studies like ours. Our study is based on cross-sectional data, which limits inferences about the progression of the cognitive connectome across the different clinical stages. As such, these findings should be considered descriptive and hypothesis-generating. Therefore, future longitudinal or interventional studies with pathologically confirmed patients will be crucial to clarify the trajectory of network alterations and their potential clinical relevance.

## Conclusion

In conclusion, our study extends the findings from previous univariate (for a review^30^ and multivariate cognitive studies in DLB^18^, demonstrating that cognitive connectome alterations are already present in the prodromal stage of MCI-LB. These alterations are still modest but suggest a reduced network segregation that translates into a loss of cognitive specialization. The comparison between the two MCI groups uncovered the role of memory retrieval in differentiating MCI-LB from MCI-AD. The comparison of MCI-LB with DLB showed numerous significant differences, indicating a more pronounced loss of cognitive specialization at the dementia stage. Once the observed alterations are confirmed in longitudinal studies, insights from cognitive connectomes may help improve the challenging clinical detection of MCI-LB patients. Moreover, our findings may provide preliminary insights into cognitive profiles along the progression of the disease. A better understanding of associations among cognitive measures in MCI-LB may guide future research on key cognitive processes as targets for cognitive stimulation^15^, before the more developed and overt cognitive impairments found in DLB.

## Methods

### Participants

For this cross-sectional study, we obtained data from the National Alzheimer’s Coordinating Center (NACC)^32^, collected across 32 Alzheimer’s Disease Research Centers in the United States between March 2015 and May 2021. We included patients aged ≥ 45 years and diagnosed with MCI-LB, MCI-AD, or DLB. Clinical diagnoses were established by the clinician’s judgement according to the available clinical diagnostic criteria at the evaluation date. Specifically, MCI-LB and DLB diagnoses were based on the McKeith criteria^2,5^, while MCI-AD diagnosis was based on the NIA-AA criteria^33,34^. The Clinical Dementia Rating (CDR) scale was used to assess clinical severity^35^. Additionally, we included a HC group who showed no cognitive impairment on clinical assessment. The included DLB and HC groups are identical to those used in our previous publication^18^.

Participants were only included if they had available data on all cognitive measures selected to construct the cognitive connectomes (see next section). Participants with a clinical history of bipolar disorder, schizophrenia, delusional disorder, craniocerebral trauma, stroke, substance abuse, and uncorrected vision or hearing problems were excluded from this study. All participants gave written informed consent prior to the study, and local Institutional Review Boards approved the study.

### Cognitive measures and construction of cognitive connectomes

The neuropsychological protocol of the NACC database is fully described elsewhere^32^. We included 25 cognitive measures covering six cognitive domains, i.e., visuoconstructive functions, visual and verbal episodic memory, processing speed, attention, executive functions, language, and orientation. The assignment of each cognitive measure to cognitive domains was based on standard classifications and previous studies^9,36,37^ (see Table 2). We calculated the domain-specific performance for each group by z-scoring cognitive measures using the HC group as the reference, and averaging z-scores for each domain as detailed in Table 2. Variables with missing values in more than 15% of the patients were excluded. These were a total of four variables, including the total score, commission errors, and correct lines in the Trail Making Test-Part B, as well as the number of correct responses in the MINT after cues.

Before constructing the cognitive connectomes, we carefully inspected the distribution and nature of all 25 cognitive measures. To facilitate subsequent statistical analysis and interpretation of results, we inverted cognitive measures when necessary, thus ensuring that higher scores always indicated a better performance, and transformed skewed measures. This procedure allows easier interpretability and avoids negative correlations that would distort network topology during the subsequent binarization. To account for the effect of age, sex, and education on cognitive performance, we removed their effects on all cognitive measures by using multiple linear regression or logistic regression as detailed elsewhere^38^. Subsequently, we inspected the normal distribution of the cognitive measures once more. During this inspection, we noticed that one measure was not normally distributed. Therefore, we applied Spearman correlation to define the edges of the cognitive connectomes. As one cognitive measure (i.e., repetition errors in phonemic fluency) barely correlated with the rest of the measures, we excluded it from further statistical analysis since we aimed to investigate unfragmented cognitive connectomes. The cognitive connectomes for MCI-LB, HC, MCI-AD, and DLB groups were constructed by pair-wise Spearman correlations between the remaining 24 cognitive measures (Table 2).

Following previous cognitive connectome studies, we included positive and negative correlations in the correlation matrices while excluding self-connections^12,17^. In line with common procedure, we binarized correlation matrices across a range of network densities (fraction of connected edges), based on the cognitive connectome of the HC group. As the strong correlations between cognitive measures derived from the Montreal Cognitive Assessment (MoCA) increased the threshold density at which other nodes became connected, we excluded MoCA measures to determine the range of network densities. Within the selected density range, nodes tended to be connected to at least one other node (densities > 10%), and the connectome exhibited a non-random topology (densities < 30% with small-worldness > 1). Next, we included all MoCA measures again and confirmed the suitability of this density range for the complete connectome in all four study groups. Specifically, the suitability of the 10%−30% range of densities was confirmed by ensuring that there were generally no disconnected connectomes or random topologies. Of note, in the connectome of DLB patients, the small-world index fell below 1 (indicating a random topology) at densities < 30%.

**Table 2:** List of cognitive measures included in the cognitive connectomes.

Cognitive measures	Cognitive domains
Benson complex figure copy	Visuoconstructive functions
MoCA cube and clock items	
Benson complex figure delayed	Visual and verbal episodic memory
Benson complex figure recognition	
Craft story 21 Immediate paraphrase	
Craft story 21 delayed paraphrase	
MoCA immediate	
MoCA delayed	
MoCA category cue	
MoCA recognition	
Verbal fluency phonemic Verbal fluency phonemic intrusions (inverted) Verbal fluency semantic	Executive functions
TMT commissions (inverted)	
MoCA trail	
MoCA abstraction	
Digit span forward Digit span backward	
TMT part A seconds (inverted)	Processing speed/attention
MoCA calculation	
MoCA letter A	
MINT	Language
MoCA repetition	
MoCA orientation items	Orientation

For most cognitive measures in the table, performance reflects the number of correct elements, otherwise specified in the table (i.e., intrusions, commissions, and seconds). The table also specifies which measures were inverted. Abbreviations: MINT, Multilingual Naming Test; MoCA, Montreal Cognitive Assessment; TMT, Trail Making Test.

### Network measures

To investigate the MCI-LB cognitive connectome, we computed graph-theoretical network measures to reflect centrality (importance of cognitive measure), integration (communication across cognitive measures), and segregation (specialisation of cognitive measures) that were demonstrated to be stable in previous studies^39^ and have previously been used to investigate cognitive connectomes^9,18^. Specifically, we calculated the average strength (a measure of the magnitude of correlations), global efficiency (a measure of integration), transitivity (a measure of segregation), and local efficiency (a measure of segregation) on the global network level^40^. Additionally, we calculated the strength (a measure of centrality) and participation (measures of centrality), global efficiency, and local efficiency on the nodal network level^40^. All network measures are fully described elsewhere^18^. The global and nodal strength measures were calculated on weighted networks, while all other remaining measures were calculated on binary networks.

### Statistical analysis

We used ANOVAs to assess group differences in demographic and clinical variables. Additionally, we applied ANCOVAs including age, sex, and education as covariates to compare performances across groups across six cognitive domains. Statistical significance was set to *p* < 0.05 in all these analyses.

Between-group comparisons of network measures were conducted using 1000 nonparametric permutations across the defined range of network densities from 10% to 30%, in steps of 1%. Only global measures that showed differences across ≥ 5 consecutive densities were considered significant, thus representing stable differences across the range of densities^41^. Additionally, we demonstrated the stability of the significant differences in global measures by repeating analyses using five iterations of a leave-five-out approach, therefore each time excluding five random participants from each diagnostic group. Since the diagnostic groups were different in size, we also replicated the significant differences in global measures by randomly selecting 88 patients with MCI-AD, DLB, or HC, to match the size of the smaller target group MCI-LB (*n* = 88). For nodal measures, we additionally applied a false discovery rate (FDR) adjustment at *p* ≤ 0.05 (two-tailed)^42^. We report nodal results at the median density (20%)^9,18,43^, while ensuring that median results were representative of the results for adjacent densities. Again, we demonstrated the stability of the significant differences in nodal measures by repeating analyses using five iterations of a leave-five-out approach, each time excluding five random participants from each diagnostic group.

Network analyses were performed using the BRAPH 1.0.0 interface^20^ in Matlab R2024a, following procedures outlined in the publicly available manual, while ANOVAs and ANCOVAs were carried out using R Studio with the ULLRToolbox and SPSS version 25.0.

## Data Availability

The data used in this study are from the NACC. Data was available to the authors through https://naccdata.org/. For further information upon data availability, contact Daniel Ferreira.
